# scDAU: a disentangled representation learning method for cross-modal translation in single-cell multi-omics data

**DOI:** 10.1093/bioinformatics/btag610

**Published:** 2026-08-14

**Authors:** Jialiang Xue, Xiangmei Cao, Junlei Zhou, Yaowei Cao, Fangyuan Shi, Fang Du, Zhenhua Yu

**Affiliations:** School of Information Engineering, Ningxia University, Yinchuan, Ningxia 750021, China; Basic Medical School, Ningxia Medical University, Yinchuan, Ningxia 750001, China; School of Information Engineering, Ningxia University, Yinchuan, Ningxia 750021, China; School of Information Engineering, Ningxia University, Yinchuan, Ningxia 750021, China; School of Information Engineering, Ningxia University, Yinchuan, Ningxia 750021, China; School of Information Engineering, Ningxia University, Yinchuan, Ningxia 750021, China; School of Information Engineering, Ningxia University, Yinchuan, Ningxia 750021, China

## Abstract

**Motivation:**

Cross-modal translation enables reconstruction of missing modalities in single-cell multi-omics data, supporting integrative analyses of cellular heterogeneity and regulatory relationships. However, existing methods often struggle to disentangle shared biological signals from modality-specific variation and to generalize across datasets.

**Results:**

We present scDAU, a deep learning framework that combines conditional diffusion-based feature regularization with multi-scale cross-modal translation networks. scDAU employs a feature decoupling strategy to separate shared semantic representations from modality-specific components, followed by U-Net-based architectures for accurate bidirectional translation between modalities. Across multiple benchmark datasets, scDAU outperforms existing methods in both within-dataset and cross-dataset settings, as well as in predicting modalities for previously unseen cell types. The framework further generalizes to transcriptome–proteome translation, demonstrating flexibility across diverse multi-omics contexts. Application to a human glioblastoma dataset showed that scDAU preserves cell-type-specific gene expression and chromatin accessibility patterns, supporting downstream analyses such as marker identification and functional enrichment. Overall, scDAU provides a robust and extensible approach for cross-modal translation.

**Availability:**

The source code of scDAU is available at https://github.com/zhyu-lab/scdau and https://doi.org/10.5281/zenodo.19303337.

## 1 Introduction

Recent advances in single-cell multi-omics technologies have enabled simultaneous measurement of multiple modalities in the same cell, such as gene expression with chromatin accessibility (e.g. SNARE-seq, sci-CAR, SHARE-seq) or surface protein abundance (e.g. CITE-seq, REAP-seq). These paired measurements provide integrative, multi-dimensional views of cellular states, facilitating accurate characterization of regulatory interactions, cellular heterogeneity, and disease mechanisms (Drost *et al.* 2024).

Despite these advances, experimental multi-omics profiling remains technically challenging due to increased complexity, elevated technical noise, high costs, and limitations in throughput and cross-sample comparability (Zhang *et al.* 2022). At the same time, large volumes of high-quality single-modality data, such as scRNA-seq and scATAC-seq data, have been generated using conventional single-omics technologies, yet remain underutilized due to the lack of complementary modalities (Tabula Sapiens Consortium* *et al.* 2022, Elmentaite *et al.* 2022, Ge *et al.* 2025). This creates a pressing need for computational approaches that can effectively integrate heterogeneous data or infer missing modalities. In this context, robust cross-modal translation has emerged as a promising strategy to reconstruct unmeasured modalities from available data, thereby reducing experimental costs and expanding the utility of existing datasets.

A variety of deep learning approaches have been developed for single-cell cross-modal translation, which can be broadly categorized into three groups. First, latent space alignment-based methods learn shared or coordinated representations across modalities and perform translation through reconstruction or alignment constraints. For instance, cTP-net (Zhou *et al.* 2020) maps denoised scRNA-seq data to protein abundance using multi-branch neural networks; BABEL (Wu *et al.* 2021) and scMM (Minoura *et al.* 2021) employ dual autoencoders or mixture-of-experts variational autoencoders (VAEs) to learn shared latent spaces for scRNA-seq and scATAC-seq data; Polarbear (Zhang *et al.* 2022), CLUE (Tu *et al.* 2022), MultiVI (Ashuach *et al.* 2023), UnitedNet (Tang *et al.* 2023), and JAMIE (Cohen Kalafut *et al.* 2023) refine latent fusion, partial-modality inference, or joint representation learning through VAEs or autoencoder-based architectures. Second, architecture-enhanced models introduce specialized network designs or training strategies to improve translation performance. For example, sciPENN (Lakkis *et al.* 2022) combines feed-forward and recurrent networks for RNA-to-protein translation, and scMOG (Lan *et al.* 2023) integrates adversarial training to generate ATAC profiles from RNA embeddings. Third, generative approaches, including adversarial and diffusion-based models, have been proposed to better capture complex cross-modal distributions and enable missing-modality generation. scButterfly (Cao *et al.* 2024) and scCross (Yang *et al.* 2024) leverage dual-aligned VAEs, generative adversarial networks (GANs), and neighborhood-based alignment to enhance translation robustness, while scPair (Hu and Quon, 2024) and scCobra (Zhao *et al.* 2025) integrate autoencoders with adversarial learning for bidirectional translation and batch effect correction. More recently, scDCT (Zhou *et al.* 2025) employs modality-specific encoders coupled with conditional diffusion models to achieve high-quality cross-modal translation.

While these methods demonstrate promising performance in cross-modality translation, they often rely on direct latent mapping and deterministic reconstruction, which may limit their ability to capture the intrinsic complexity and variability of multi-omics data. In addition, the learned representations frequently entangle cross-modally shared biological signals with modality-specific variation, including technical noise. Insufficient disentanglement of these components can impair both translation accuracy and generalization.

To address these challenges, we present scDAU, an effective and extensible framework for single-cell cross-modal translation. Unlike scDCT, which employs conditional diffusion directly for cross-modal translation in a latent space, scDAU utilizes conditional diffusion as a feature regularization mechanism and explicitly disentangles shared and modality-specific representations. By integrating conditional diffusion-based feature regularization with multi-scale translation networks, scDAU enables robust bidirectional translation between modalities. Furthermore, by combining feature decoupling with U-Net–based architectures, scDAU improves both global structural consistency and local feature fidelity in reconstructed data.

We systematically evaluated scDAU across multiple real datasets under diverse scenarios, including within-dataset translation, cross-dataset generalization, and prediction for unseen cell types. In all settings, scDAU demonstrated consistent improvements over existing methods. Furthermore, the framework generalizes to additional modalities, including transcriptome–proteome translation. Application to a human glioblastoma dataset showed that scDAU preserves cell-type-specific gene expression and chromatin accessibility patterns, supporting downstream analyses such as marker identification and functional enrichment. Together, these results demonstrated that scDAU provides a robust and practical approach for cross-modal translation, facilitating integrative analysis and reuse of single-cell multi-omics data.

## 2 Materials and methods

### 2.1 scDAU workflow

**Figure 1:**
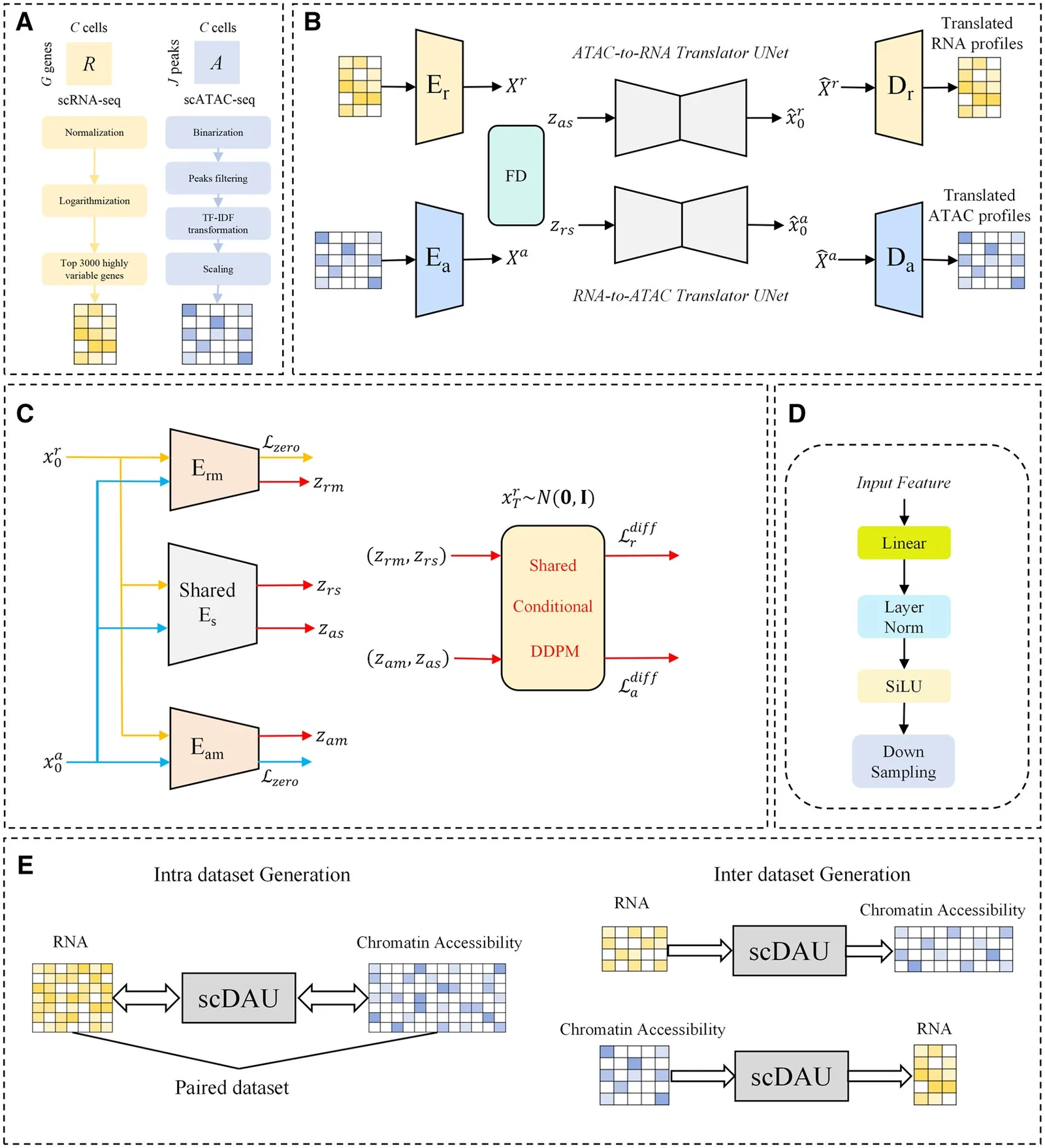
Overview of scDAU framework. (A) Preprocessing pipelines for scRNA-seq and scATAC-seq data. (B) Core workflow of scDAU. Paired scRNA-seq and scATAC-seq data are first processed by two independent autoencoders for dimensionality reduction. Shared biological signals are then disentangled through a feature decoupling module. Based on the extracted shared features, cross-modal translation is performed using two U-Net based networks. Finally, the translated representations are reconstructed back to the original data space using modality-specific decoders. (C) Feature decoupling module. This module consists of an RNA-specific encoder $E_{\text{rm}}$, an ATAC-specific encoder $E_{\text{am}}$, and a shared encoder $E_{s}$. The decoupling process is regularized via a shared conditional diffusion model to enforce effective separation of shared and modality-specific signals. (D) Encoder layer of the U-Net network architecture. Input features are sequentially processed through linear transformation, layer normalization, and SiLU activation function for downsampling. The upsampling layer adopts a symmetric architecture. (E) Evaluation scheme of scDAU. First, within-dataset performance is assessed, where training and testing sets are drawn from the same data distribution. Second, the trained scDAU model is evaluated for its ability to generate missing modalities on new datasets containing only a single modality (either scRNA-seq or scATAC-seq).

The architecture of scDAU is depicted in Fig. 1, and its workflow comprises three main stages. Stage 1 performs nonlinear dimensionality reduction to capture complex biological variation in single-cell data (Fig. 1B). In Stage 2, scDAU introduces an RNA-specific encoder $E_{\text{rm}}$, an ATAC-specific encoder $E_{\text{am}}$, a shared semantic encoder $E_{s}$, and a shared conditional diffusion process that serves as a feature space regularizer to disentangle modality-specific features from cross-modally shared semantic representations (Fig. 1C). In Stage 3, based on the representations learned by $E_{s}$, two cross-modal U-Nets (ATAC-to-RNA and RNA-to-ATAC) are trained to enable bidirectional translation (Fig. 1B). The translated outputs are finally projected back to the original data spaces using modality-specific decoders.

### 2.2 Data preprocessing

As shown in Fig. 1A, for scRNA-seq data, raw read counts were first normalized by sequencing depth to ensure comparability across cells, a log(x+1) transformation was then applied to stabilize variance. To focus the analysis on biologically informative signals, the top 3000 highly variable genes (HVGs) were selected, resulting in a C×G gene expression matrix *R*, where *C* is the number of cells and *G* is the number of selected genes.

For scATAC-seq data, peaks detected in at least 0.5% of cells were retained to remove low-confidence or spurious signals. The resulting peak-by-cell matrix was binarized to indicate the presence or absence of chromatin accessibility. We then employed Term Frequency-Inverse Document Frequency (TF-IDF) (Zandigohar and Dai 2022) weighting and global max normalization to process the data. This yielded a C×J peak matrix *A*, where *J* denotes the number of selected accessible regions.

### 2.3 Dimensionality reduction with autoencoders

To address the challenges posed by the high dimensionality of single-cell data in multi-modal translation tasks, we employed modality-specific autoencoders to perform dimensionality reduction. The encoders $E_{r}$ and $E_{a}$ project the original RNA and ATAC inputs into a low-dimensional latent space of 256 dimensions. The decoders, $D_{r}$ and $D_{a}$, reconstruct the original data from these latent representations. Detailed structures of the autoencoders are provided in Supplementary Methods, available as supplementary data at *Bioinformatics* online.

When training the autoencoders, the mean squared error (MSE) loss was used for scRNA-seq data:


(1) $$L_{r}=\frac{1}{C}\sum_{i=1}^{C}\|R_{i}-D_{r}(E_{r}(R_{i})){\|}_{2}^{2}$$

while the binary cross-entropy (BCE) loss was adopted for scATAC-seq data:


(2) $$L_{a}=-\frac{1}{C}\sum_{i=1}^{C}\left[A_{i}\;\text{log}\;{\hat{A}}_{i}+(1-A_{i})\;\text{log}(1-{\hat{A}}_{i})\right]$$

where ${\hat{A}}_{i}=D_{a}(E_{a}(A_{i}))$. After training, the encoders $E_{r}$ and $E_{a}$ generate low-dimensional representations $X^{r}$ and $X^{a}$ for the RNA and ATAC modalities, respectively.

### 2.4 Feature decoupling for cross-modal bidirectional translation

To effectively disentangle modality-specific variation from cross-modally shared biological signals, we introduced a feature decoupling module composed of three dedicated encoders (Fig. 1C): an RNA-specific encoder $E_{\text{rm}}$, an ATAC-specific encoder $E_{\text{am}}$, and a shared semantic encoder $E_{s}$. All three encoders were implemented as multilayer perceptrons (MLPs), each consisting of an input layer, a hidden layer with LeakyReLU activation, and a linear output layer that produces latent representations.

Given the low-dimensional embeddings $X^{r}$ and $X^{a}$ obtained from $E_{r}$ and $E_{a}$, the encoders $E_{\text{rm}}$ and $E_{\text{am}}$ extract modality-specific features $Z_{rm}$ and $Z_{am}$, respectively. In parallel, the shared encoder $E_{s}$ processes the same inputs to produce shared semantic representations $Z_{rs}$ and $Z_{as}$, which capture modality-invariant biological information common across omics layers. To guide the encoders towards learning high-quality and well-disentangled representations, the feature decoupling module was trained under two complementary constraints. First, a shared conditional diffusion model was employed to regularize feature extraction by reconstructing the original data from the extracted latent features. Second, a zero-loss constraint was imposed to explicitly encourage the separation of modality-specific information from the shared latent space. The zero loss was defined as follows:


(3) $$L_{\text{zero}}=\frac{1}{C}\sum_{i=1}^{C}{\left\|E_{\text{rm}}\left(X_{i}^{a}\right)\right\|}_{1}+{\left\|E_{\text{am}}\left(X_{i}^{r}\right)\right\|}_{1}$$

The zero-loss acts as a sparsity-inducing regularizer by minimizing the outputs of modality-specific encoders when processing inputs from the opposite modality. Consequently, features that remain non-zero under this constraint are inherently modality-specific. This mechanism further regularizes the shared encoder to exclude modality-specific signals and focus on capturing shared biological information.

### 2.5 Feature regularization with conditional diffusion

We adopted the Denoising Diffusion Probabilistic Model (DDPM) (Ho *et al.* 2020) as the generative backbone to constrain the feature disentanglement process. DDPM is a probabilistic generative framework that defines a forward diffusion process, in which Gaussian noise is gradually added to the data, and a reverse denoising process, in which the model learns to recover the original signal. The forward diffusion process is defined as:


(4) $$q\left(x_{t}|x_{t-1}\right)=N\left(x_{t};\sqrt{\alpha_{t}}x_{t-1},\left(1-\alpha_{t}\right)I\right)$$

where t∈{1,…,T} denotes the diffusion step, *T* is the total number of steps, and $\left\{\alpha_{t}\right\}$ is a predefined noise schedule controlling the variance added at each step. $x_{t}$ can be directly sampled from the original data $x_{0}$:


(5) $$x_{t}=\sqrt{{\bar{\alpha }}_{t}}x_{0}+\sqrt{1-{\bar{\alpha }}_{t}}\epsilon$$

where $\epsilon_{t}∼N(0,I)$ and ${\bar{\alpha }}_{t}=\prod_{s=1}^{t}\alpha_{s}$ is the cumulative signal retention factor.

In the reverse denoising process, the model estimates the conditional distribution:


(6) $$p_{\theta }\left(x_{t-1}|x_{t}\right)=N\left(x_{t-1};\mu_{\theta }\left(x_{t},t\right),\Sigma_{\theta }\left(x_{t},t\right)\right)$$

with $\mu_{\theta }$ and $\Sigma_{\theta }$ being parameterized by a neural network with parameters θ. Following standard practice, the diffusion model is trained by minimizing a simplified noise prediction objective:


(7) $$L_{\text{diff}}=E_{x_{0},t,\epsilon_{t}}\left[\|\epsilon_{\theta }(x_{t},t)-\epsilon_{t}{\|}_{2}^{2}\right]$$

where $\epsilon_{\theta }(\cdot )$ represents the noise estimation network implemented using a U-Net architecture.

To enforce meaningful feature disentanglement, we employed a conditional diffusion strategy in which the generation process is guided by both modality-specific variations and shared biological signals. For RNA reconstruction, a conditional DDPM was used to generate ${\hat{X}}^{r}$ conditioned on [$Z_{rm}$, $Z_{rs}$], and the objective was defined as:


(8) $$L_{r}^{\text{diff}}=E_{x_{0}^{r},t,\epsilon_{t}}\left[\|\epsilon_{\theta }(x_{t}^{r},t|z_{rm},z_{rs})-\epsilon_{t}{\|}_{2}^{2}\right]$$

where $x_{0}^{r}$ represents RNA embedding, $z_{rm}$ and $z_{rs}$ are the modality-specific and shared RNA features, respectively.

Similarly, ATAC feature extraction was constrained using the same conditional diffusion model, optimized by:


(9) $$L_{a}^{\text{diff}}=E_{x_{0}^{a},t,\epsilon_{t}}\left[\|\epsilon_{\theta }(x_{t}^{a},t|z_{am},z_{as})-\epsilon_{t}{\|}_{2}^{2}\right]$$

where $x_{0}^{a}$ denotes ATAC embedding, $z_{am}$ and $z_{as}$ are the corresponding modality-specific and shared features.

Through these reconstruction constraints, the conditional DDPM regularizes the feature extraction process, ensuring that both modality-specific and shared representations are informative and biologically meaningful.

### 2.6 Overall objective for feature disentanglement

The feature decoupling objective jointly optimizes the diffusion reconstruction losses and the zero-loss constraint that enforces separation between modality-specific variations and shared biological signals. The overall loss function was defined as:


(10) $$L=\lambda_{1}(L_{r}^{\text{diff}}+L_{a}^{\;\text{diff}})+\lambda_{2}L_{\text{zero}}$$

where $\lambda_{1}$ and $\lambda_{2}$ are weighting hyperparameters controlling the trade-off between diffusion reconstruction fidelity and feature disentanglement. By default, $\lambda_{1}$ = 0.5 and $\lambda_{2}$ = 1.0.

### 2.7 U-Nets for cross-modal translation

Given the shared biological features $Z_{rs}$ and $Z_{as}$, scDAU constructed two cross-modal U-Net networks (Ronneberger *et al.* 2015) to achieve bidirectional translation between modalities. Each U-Net adopted a symmetric four-layer encoder-decoder architecture with skip connections, enabling multi-scale feature fusion and progressive semantic alignment. This hierarchical structure preserves both global semantic information and local details, reducing information loss compared to direct one-step mapping and improving translation robustness.

As illustrated in Fig. 1D, each encoder layer consists of a linear transformation followed by layer normalization, a SiLU activation, and a downsampling block. The upsampling path mirrors the downsampling structure and incorporates skip connections to fuse features from corresponding encoder layers, ultimately enabling high-fidelity reconstruction in the target modality.

For RNA-to-ATAC translation, the U-Net takes $Z_{rs}$ as input and generates a low-dimensional ATAC representation ${\hat{X}}^{a}$, optimized using an MSE loss against the true ATAC embedding $X^{a}$. Similarly, the ATAC-to-RNA U-Net maps $Z_{as}$ to ${\hat{X}}^{r}$, trained by minimizing the MSE with respect to the RNA embedding $X^{r}$. After training, the translated embeddings ${\hat{X}}^{a}$ and ${\hat{X}}^{r}$ were decoded by the modality-specific decoders $D_{a}$ and $D_{r}$, respectively, yielding cross-modally translated data in the original feature spaces.

### 2.8 Performance evaluation metrics

To assess the performance of scDAU, we quantified the agreement between the generated data and the corresponding experimental measurements. For scRNA-seq data, the Pearson Correlation Coefficient (PCC) is employed to evaluate the linear correlation between predicted and observed gene expression profiles. PCC is widely used to quantify the co-variation patterns in gene expression (Buenrostro *et al.* 2015). For scATAC-seq data, treating the original measurements as ground truth, the area under the receiver operating characteristic curve (AUROC) is computed to assess the accuracy of the generated accessibility profiles. Details regarding calculation of these two metrics are provided in the Supplementary Methods.

### 2.9 Datasets and baseline methods

To comprehensively evaluate scDAU, we employed four publicly available paired single-cell multi-omics datasets spanning diverse platforms, species, and tissue types: (1) 10X-PBMC: peripheral blood mononuclear cells from healthy donors profiled with Cell Ranger ARC 1.0.0 for simultaneous scRNA-seq and scATAC-seq, comprising 9631 cells across 19 cell types; (2) BMMC: a 10X Genomics benchmark dataset with 13 samples from 10 donors, totaling over 69 000 cells; (3) MA: adult mouse tissues (skin, brain, and lung) profiled via SHARE-seq, containing 32 231 cells across 4 major clusters; (4) Chen: adult mouse brain tissue profiled using SNARE-seq, consisting of 9190 cells across 22 clusters. Additionally, we applied scDAU to an in-house human glioblastoma dataset, consisting of 9744 cells across 8 cell types, to assess its performance in tumor contexts. More details of the human glioblastoma dataset are given in the Supplementary Methods. For cross-dataset generation experiments, models pre-trained on the PBMC and MA datasets were applied to two independent test sets: a PBMC set with 11 331 cells and an MA set with 5692 cells. scDAU was benchmarked against six state-of-the-art single-cell cross-modal translation methods: BABEL (Wu *et al.* 2021), scMM (Minoura *et al.* 2021), JAMIE (Cohen Kalafut *et al.* 2023), scMOG (Lan *et al.* 2023), scButterfly (Cao *et al.* 2024), and scDCT (Zhou *et al.* 2025). All baseline methods were run using the optimal hyperparameters reported in their respective publications to ensure fair comparisons. Implementation details of scDAU are provided in the Supplementary Methods.

## 3 Results

### 3.1 scDAU performs well on within-dataset generation

To assess the quality of RNA and ATAC data generated by scDAU, cross-modal translation experiments were conducted within each dataset using a five-fold cross-validation strategy. For each fold, 80% of the dataset was used for training, with 16% of the training data further held out as a validation set, and the remaining 20% reserved for testing.

**Figure 2:**
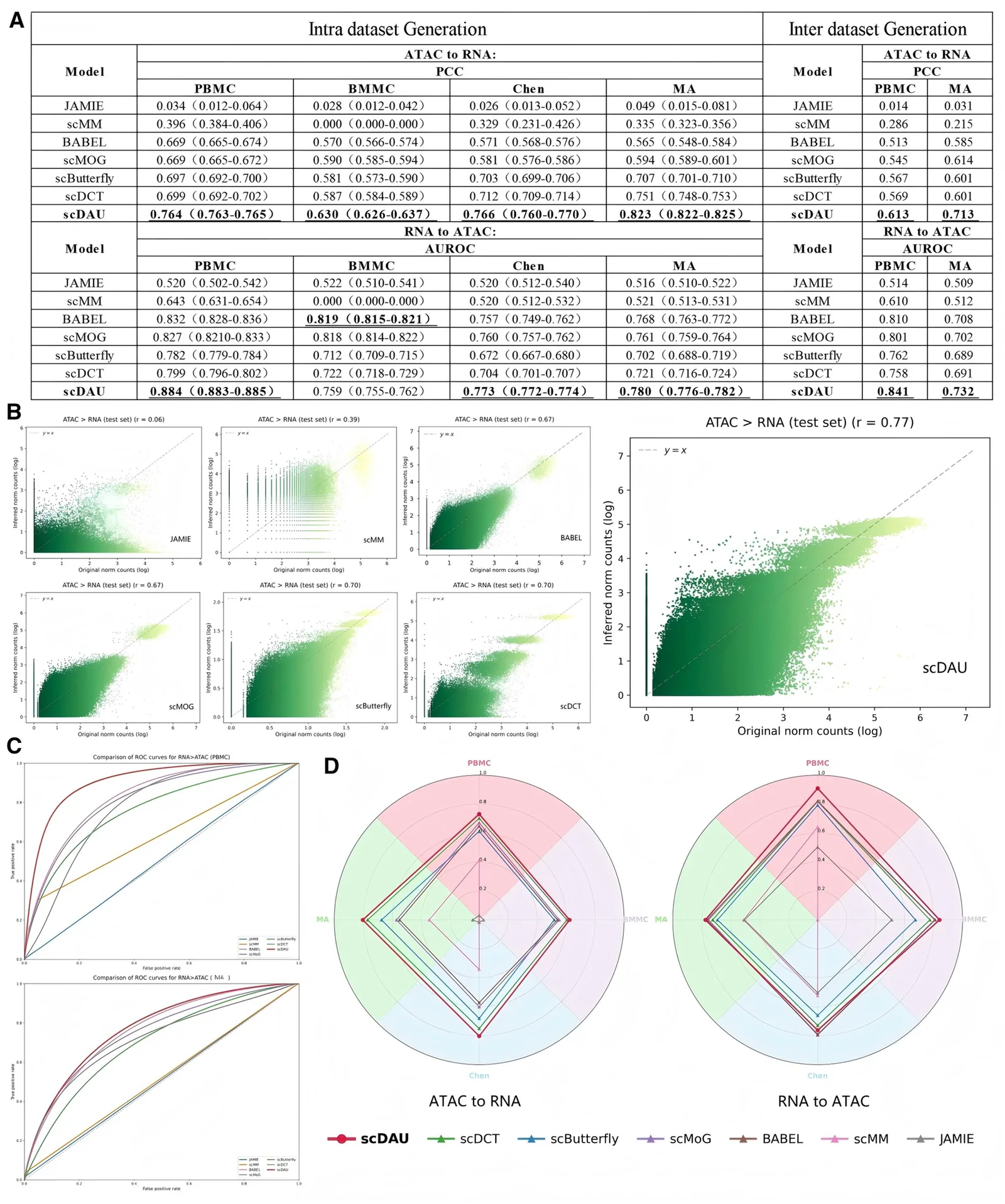
Performance evaluation of scDAU across multiple datasets. (A) Quantitative performance comparison under both within-dataset and cross-dataset evaluation settings. scMM encounters data processing errors on the BMMC dataset, therefore fails to produce valid outputs. (B) Visualization of ATAC-to-RNA translation performance on the PBMC dataset. The plot reports the Pearson correlation coefficient (PCC) between the generated RNA profiles and the corresponding raw RNA data in the test set. (C) Visualization of RNA-to-ATAC translation performance. The area under the receiver operating characteristic curve (AUROC) is computed by comparing the generated ATAC profiles with the raw ATAC data on the PBMC and MA datasets. (D) Performance evaluation on unseen cell types. This assesses the generative capability of scDAU when translating modalities for cell types that are completely excluded from the training data.

In the ATAC-to-RNA translation task, scDAU outperformed six baseline methods across all four datasets, as measured by the Pearson correlation coefficient. On the PBMC dataset (Fig. 2A), scDAU achieved a PCC of 0.764, approximately 6% higher than the second-best method and 10% higher than the others. Similar trends were observed in the remaining datasets, scDAU maintaining top performance, exceeding the runner-up by 4% and other methods by more than 6%. Visualization analyses further corroborated this advantage. The RNA data generated by scDAU aligned more closely with the original data (Fig. 2B), implying its robust translation capability. For the RNA-to-ATAC translation task, scDAU attained the highest AUROC scores on three out of the four datasets (0.884, 0.773, and 0.780). On the BMMC dataset, scDAU yielded an AUROC of 0.759, lower than BABEL (0.819). Nevertheless, visualization of AUROC curves (Fig. 2C and Fig. S1, available as supplementary data at *Bioinformatics* online) demonstrated that, particularly on the PBMC and MA datasets, scDAU obviously surpassed competing methods, underscoring its consistent overall performance.

Additional analysis on the PBMC and MA datasets confirmed the effectiveness of feature disentanglement (Fig. S2, available as supplementary data at *Bioinformatics* online). t-SNE visualization showed that shared features ($Z_{rs}$ and $Z_{as}$) were well aligned and primarily captured biologically meaningful structure, whereas modality-specific features ($Z_{rm}$ and $Z_{am}$) formed clearly separated clusters with limited correspondence to cell-type organization (more details in Supplementary Results). Consistently, K-means clustering revealed that only the shared features recovered the topological structure of cell types, while modality-specific features exhibited largely homogeneous clustering patterns, indicating that cell-type information was predominantly encoded in the shared latent space.

We further conducted a runtime performance comparison among all methods. The results (Supplementary Tables 1–2, available as supplementary data at *Bioinformatics* online) showed that scDAU achieved competitive training speed while maintaining relatively low RAM consumption across datasets of different scales. Detailed runtime analyses are presented in Supplementary Results.

### 3.2 scDAU enables accurate cross-dataset translation

To further investigate the generalizability and practical utility of scDAU, we performed cross-dataset generation experiments. These experiments were designed to evaluate the model’s performance when applied to entirely new datasets that may exhibit distributional shifts relative to the data used for training. The goal is to test whether scDAU could capture robust, transferable biological features that generalize beyond the original dataset. The experimental procedure involves two main steps: (1) Training on the source dataset. A scDAU model was trained using all available samples from a source dataset (e.g. PBMC or MA); (2) Application to an unseen target dataset. The fully trained model was then directly applied to perform cross-modal translation on a separate, unseen target dataset of the same biological type (a different PBMC or MA dataset). For this evaluation, two separate scDAU models were trained using the complete PBMC and MA datasets, respectively. These models were subsequently evaluated on two independent test sets: a new PBMC dataset and a new MA dataset, each representing distinct experiments with potential batch effects and subtle biological differences.

In the ATAC-to-RNA translation task, scDAU showcased better performance compared to all baseline methods (Fig. 2A). On the new PBMC dataset, scDAU was the only method for which the Pearson correlation coefficient between the generated and real RNA data exceeds 0.6. Similarly, on the new MA dataset, scDAU achieved a PCC above 0.7, representing an improvement of approximately 10% over the second-best method and obviously outperforming other competitors. For the RNA-to-ATAC translation task, scDAU achieved AUROC scores of 0.841 and 0.732 on the new PBMC and MA datasets, respectively—the highest among all methods. This suggested that scDAU not only accurately predicted gene expression from chromatin accessibility but also faithfully reconstructed chromatin landscapes from RNA data in previously unseen datasets.

### 3.3 scDAU robustly translates modalities for unseen cell types

To further evaluate scDAU’s ability to generate data for unseen cell populations, we designed a cell-type-specific generation experiment. For each dataset, cells were first partitioned into distinct clusters using the Leiden clustering algorithm (Traag *et al.* 2019). We then performed five independent evaluation rounds. In each round, one of the second- to sixth-largest clusters (by cell count) was held out as the test set, representing a completely unseen cell type during training. The remaining cells, excluding the test set, were used to train the model. For methods requiring a validation set, the largest cluster in each dataset was consistently assigned as the validation set across all rounds, ensuring a fair and consistent comparison under identical test conditions. Performance metrics were then averaged across the five rounds to produce a robust assessment.

The results were summarized in the radar chart as shown in Fig. 2D. In the ATAC-to-RNA translation task, scDAU (red) yielded the highest PCC on all four datasets, as indicated by its profile consistently occupying the outermost ring of the radar chart. For the RNA-to-ATAC translation, scDAU also surpassed competing methods on most datasets. On the Chen dataset, scDAU’s performance was slightly lower than that of scMOG (purple), which may be due to the high heterogeneity of this dataset. Overall, these results showed that scDAU possessed good cross-modal translation capability, successfully generating accurate profiles for cell types completely absent from the training data.

### 3.4 scDAU generates biologically meaningful omics data

To systematically evaluate the biological significance and downstream applicability of the data generated by scDAU, we examined whether the translated omics profiles support analytical workflows comparable to those applied to experimentally measured scRNA-seq and scATAC-seq data. Specifically, we aim to extract and validate biological signals embedded within the scDAU-generated data across multiple levels of analysis.

**Figure 3:**
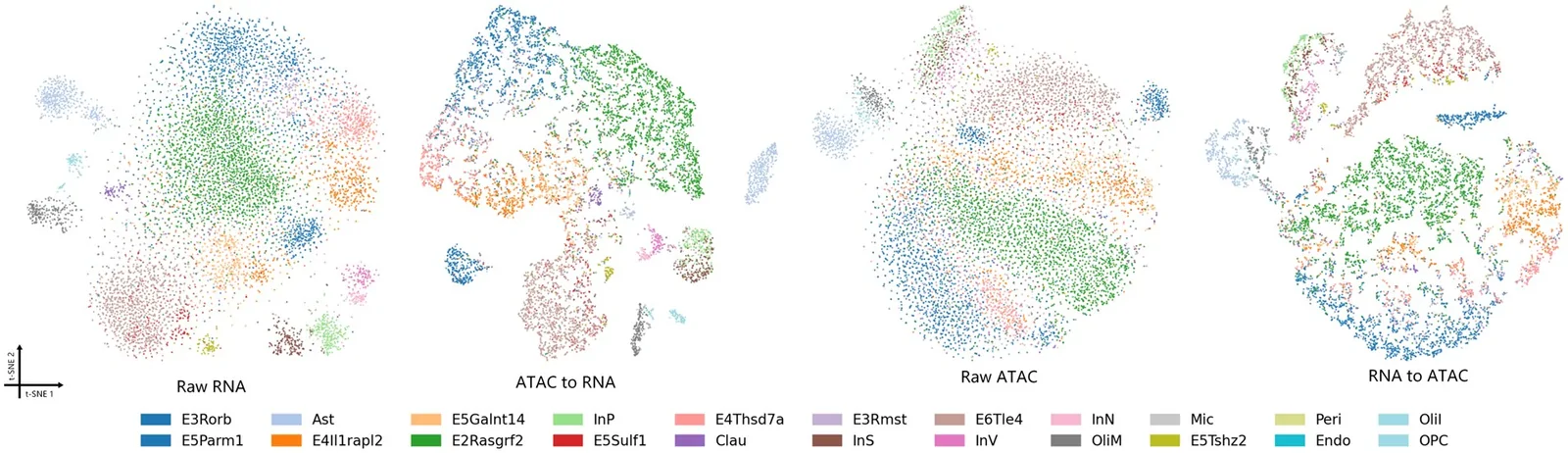
Visualization of the raw and scDAU-generated data for the Chen dataset.

We first conducted a visual assessment of the generated data. Using the Chen dataset as an example, dimensionality reduction was performed with principal component analysis (PCA), followed by visualization using the t-SNE algorithm (Van der Maaten and Hinton 2008). As shown in Fig. 3, the scDAU-generated RNA and ATAC data were projected alongside their experimentally measured counterparts. The generated data successfully recapitulated all 22 annotated cell types and exhibited spatial distributions highly consistent with those of the original data. For instance, astrocytes (Ast) formed a well-defined and isolated cluster, while inhibitory neurons subtypes (InV, InN, InS, InP) were clearly separated. These observations indicated that scDAU effectively preserved global cellular structure, cell-type composition, and inter-cell-type relationships, supporting its suitability for downstream exploratory and integrative analyses.

To assess whether scDAU preserved key biological signals during ATAC-to-RNA translation, we identified differentially expressed genes (DEGs) between cell types in both the original and translated RNA data using the non-parametric Wilcoxon rank-sum test. For each cell type, the top three DEGs were selected based on log fold-change (logFC). In the BMMC dataset (Figs S3A and 3B, available as supplementary data at *Bioinformatics* online), these genes exhibited clear cell-type-specific expression patterns. Notably, the translated RNA data retained highly variable genes, with expression profiles showing substantial overlap with those derived from the original data across most cell types. By comparing these DEGs against known marker genes curated in the CellMark 2.0 database, strong concordance with established cell-type markers was observed, such as *MME*, *AKAP12*, and *ARPP21* for lymph progenitors; *PTH2R*, *KIAA1211*, and *ADANTS3* for proerythroblasts; and *LINGO2*, *NCAM1* for natural killer (NK) cells.

We further evaluated whether scDAU-generated ATAC data retained regulatory information by analyzing differentially accessible peaks across cell types and searched for significantly enriched DNA sequence motifs within these regions. As illustrated by the seqLogo plots in Fig. S3C and 3D, motif patterns identified from the scDAU-generated data in the PBMC dataset closely matched those derived from the original experimental measurements. This high degree of consistency implied that scDAU preserved key regulatory signatures associated with transcription factor activity.

### 3.5 scDAU enables cross-modal translation to and from proteomics data

To assess the extensibility of scDAU beyond transcriptomic and epigenomic modalities, we applied it to single-cell proteomics data, exploring its ability to perform bidirectional translation between transcriptomic profiles and protein expression levels measured by antibody-derived tags (ADTs). We designed RNA-to-ADT and ADT-to-RNA translation experiments using the BMMC-CITE dataset (GEO: GSE194122). For comparison, we benchmarked scDAU against three state-of-the-art methods that are applicable to proteomics translation tasks: scDCT, scButterfly, and scMOG. All experiments followed the same five-fold cross-validation strategy used in previous evaluations, with data splits of 64% for training, 16% for validation, and 20% for testing.

**Figure 4:**
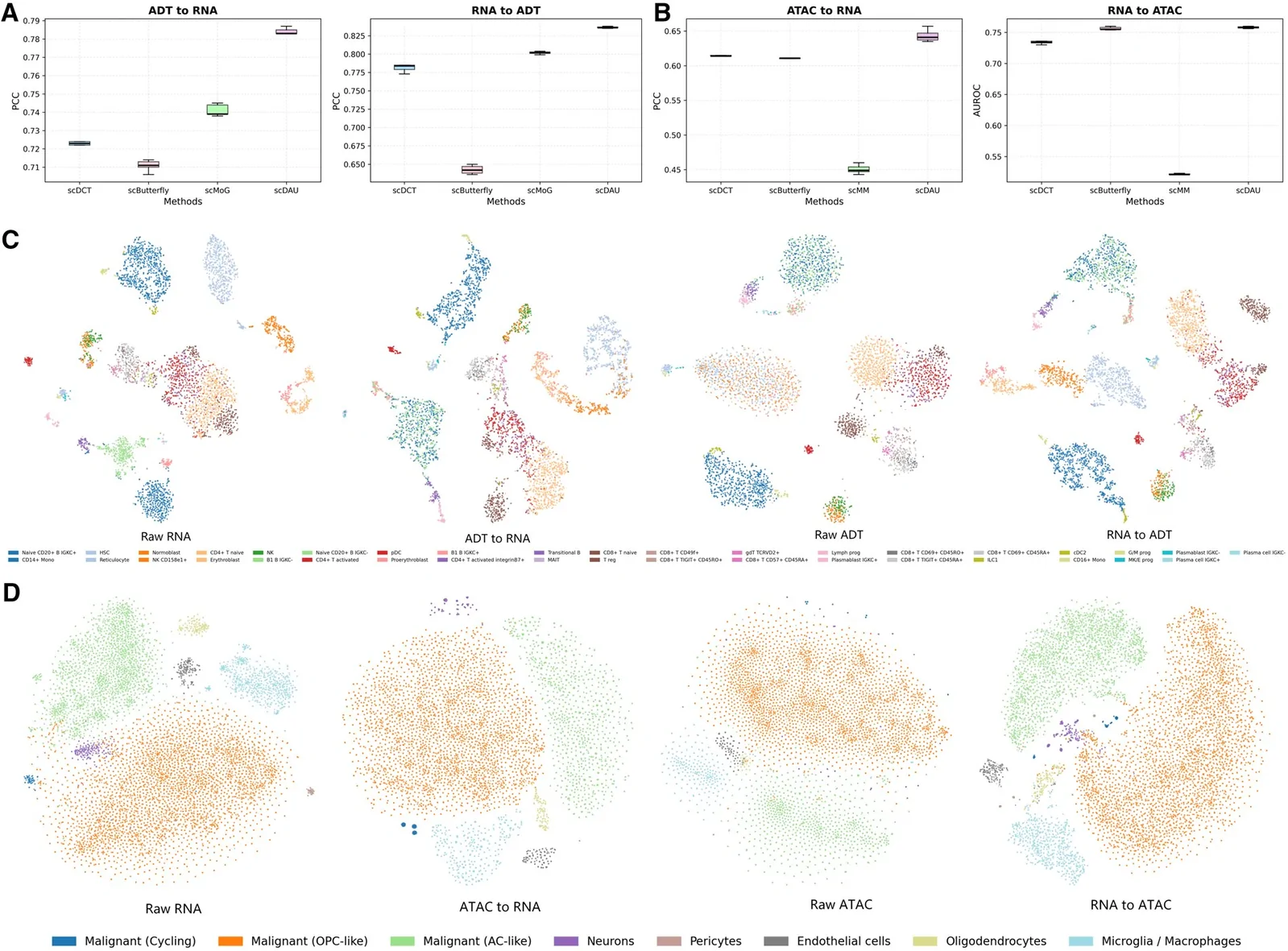
scDAU has good translation performance on the protein and human glioblastoma datasets. (A) Performance metrics on the BMMC-CITE dataset. (B) Performance metrics on the human glioblastoma dataset. scMoG and BABEL filter the entire data matrix during data preprocessing and therefore fail to generate valid outputs on this dataset. (C) Visualization of the raw and translated data on the BMMC-CITE dataset. (D) Visualization of the raw and translated data on the human glioblastoma dataset.

As summarized in Fig. 4A, scDAU yielded the highest scores in both translation directions. In the RNA-to-ADT translation task, scDAU attained a PCC of 0.782 between the generated and experimentally measured ADT profiles, representing an improvement of 4% over the second-best method, scMOG. For the ADT-to-RNA translation, scDAU delivered a PCC of 0.836, exceeding scMOG by more than 3%. Visualization analyses of the translated data (Fig. 4C) further confirmed that scDAU-generated profiles closely recapitulated both the global distribution and the local cell-type neighborhood structure observed in the original data. Notably, translation from the inherently noisier and sparser RNA space to the ADT space presents a particularly challenging task. scDAU effectively addressed this challenge by learning robust, semantically meaningful mappings between modalities.

### 3.6 scDAU recovers missing modalities in a human glioblastoma dataset

To evaluate the applicability of scDAU to complex tumor samples, we tested it on an in-house human glioblastoma dataset comprising scRNA-seq profiles from 14 671 cells and scATAC-seq profiles from 4839 cells, where the two modalities were profiled independently. The lack of paired measurements between modalities poses a substantial challenge for cross-modal translation in tumor settings. To construct paired profiles between scRNA-seq and scATAC-seq data, we adopted a stratified sampling strategy following a previous study (Cao *et al.* 2024). Briefly, we first normalized the average frequencies of cell types shared between the two modalities to estimate their relative abundances. Based on these estimated proportions, RNA and ATAC cells were randomly matched within each cell type, ensuring balanced representation in the synthesized paired dataset. This procedure resulted in a total of 9744 paired cells spanning 8 cell types. All methods were then applied to this synthesized dataset using the same training and evaluation protocol, with data splits of 64% for training, 16% for validation, and 20% for testing.

As shown in Fig. 4B, scDAU achieved a PCC of 0.643 for ATAC-to-RNA translation and an AUROC of 0.758 for RNA-to-ATAC translation. Furthermore, visualization analyses (Fig. 4D) showed that the data generated by scDAU accurately reconstruct and clearly distinguish key neural cell populations, including malignant cells, endothelial cells, and oligodendrocytes. These results confirm scDAU’s feature preservation capability and generalizability when applied to complex tumor datasets.

To validate the biological utility of scDAU-generated data, we performed Gene Ontology enrichment, marker gene validation, and transcription factor motif analysis. As shown in Fig. S4A and 4B, available as supplementary data at *Bioinformatics* online, the translated RNA faithfully recapitulated cell-type-specific functional programs from the original data, including tumor proliferation (e.g. mitotic sister chromatid segregation in cycling malignant cells), regulation of neurogenesis, inflammatory response, synaptic transmission, myelination, and vascular/stromal remodeling such as maintenance of blood-brain barrier. Marker gene analysis showed strong concordance with established cell-type markers from CellMark 2.0 (Fig. S4C and D, available as supplementary data at *Bioinformatics* online), including elevated expression of *CKAP2L* in cycling malignant cells, *CD34* in endothelial cells, and *MAL* in oligodendrocytes. Finally, motif analysis on scDAU-generated ATAC data revealed highly consistent sequence logos for key glioblastoma regulators (*PTEN*, *MYC*, *STAT3*, *SOX2*) compared to original measurements (Fig. S5, available as supplementary data at *Bioinformatics* online), with only minor quantitative differences. These results confirm that scDAU preserves core biological programs, marker signatures, and regulatory chromatin features in complex tumor data. Detailed results are provided in the Supplementary Results.

## 4 Discussion

In this study, we present scDAU, a computational framework for cross-modal translation in single-cell multi-omics data that integrates conditional diffusion-based feature regularization with multi-scale neural architectures. By explicitly disentangling shared biological signals from modality-specific variation, scDAU enables robust and accurate reconstruction of missing modalities across diverse datasets and experimental settings.

Compared with existing approaches, scDAU demonstrates consistent performance improvements in both within-dataset and cross-dataset scenarios, as well as in predicting modalities for previously unseen cell types. These results highlight the ability of the framework to generalize across datasets and to capture biologically meaningful patterns beyond training conditions. The incorporation of diffusion-based constraints and multi-scale feature learning contributes to improved stability and fidelity of the reconstructed data. Recent advances in diffusion-based generative modeling for single-cell data have similarly highlighted their effectiveness in capturing complex data distributions and improving robustness to noise and sparsity (Luo *et al.* 2024, Li *et al.* 2025).

Importantly, our results demonstrate that the performance of scDAU is sensitive to several key hyperparameters, particularly the latent space dimensionality and the weighting coefficients governing the joint optimization objective. As shown in Fig. S6, available as supplementary data at *Bioinformatics* online, a latent dimension of 256 achieves the best performance across both PBMC and MA datasets, providing an effective balance between representational capacity and generalization. Increasing the dimensionality to 512 and 1024 leads to noticeable declines in PCC and AUROC, suggesting that excessively high-dimensional latent spaces introduce redundant or noisy features that compromise model robustness. Conversely, reducing the latent dimension to 128 or 64 also degrades performance, likely due to the loss of essential biological information required for accurate reconstruction and translation.

In addition to latent dimensionality, the weighting parameters $\lambda_{1}$ and $\lambda_{2}$ play a critical role in balancing reconstruction fidelity and feature disentanglement. Under the default setting ($\lambda_{1}=0.5$, $\lambda_{2}=1.0$), scDAU achieves optimal performance (Fig. S6, available as supplementary data at *Bioinformatics* online). When $\lambda_{1}$ increases, performance consistently declines across datasets, indicating that excessive emphasis on reconstruction weakens the constraints imposed by the feature decoupling module, leading to insufficient separation between modality-specific and shared semantic features. This, in turn, hinders effective cross-modal translation. Conversely, overly small values of $\lambda_{1}$ also reduce performance, as weakened reconstruction constraints lead to lower-quality latent representations.

Furthermore, ablation analyses (Fig. S7, available as supplementary data at *Bioinformatics* online) provide additional insights into the contributions of key architectural and training design choices. Replacing a conventional linear decoder with a conditional diffusion decoder improves translation accuracy, demonstrating the superior expressive capacity of diffusion-based reconstruction mechanisms. In addition, separating feature decoupling and modality translation into distinct training stages results in more stable and reliable performance compared to joint optimization (details are provided in Supplementary Results, available as supplementary data at *Bioinformatics* online), suggesting that these components exhibit different sensitivities to training dynamics.

Beyond methodological improvements, scDAU facilitates downstream analyses by preserving key biological signals, including cell-type-specific gene expression patterns and chromatin accessibility landscapes. Application to a glioblastoma dataset highlights the practical utility of scDAU in complex biological systems characterized by substantial cellular heterogeneity and incomplete measurements. More broadly, the ability to infer missing modalities from existing data supports the reuse of large-scale single-modality datasets and enables more comprehensive multi-omics analyses.

Despite these advantages, scDAU has several limitations that warrant further investigation. First, its reliance on autoencoder-based dimensionality reduction implies that translation performance depends on the quality of learned latent representations. Although tighter integration between dimensionality reduction and translation may improve performance, it could also introduce additional optimization challenges. Second, the current framework is primarily designed for bimodal translation; extending it to multiple omics layers would require access to more complex multi-way paired datasets and more scalable modeling strategies. Third, in cross-dataset experiments, scDAU performs well on datasets generated from the same platform; however, its cross-platform translation ability has not been systematically evaluated. Such settings are more challenging due to substantial technical and sensitivity differences between platforms. Finally, while scDAU can handle partially unpaired data via stratified sampling-based pairing, it is not designed for fully unpaired translation scenarios. Future work will explore weakly supervised strategies to address unpaired multi-omics translation, which remains an important challenge in the field (Yu *et al.* 2023).

In summary, scDAU provides a robust and extensible solution for cross-modal translation in single-cell multi-omics data.

## Supplementary Material

btag610_Supplementary_Data

## Data Availability

The PBMC dataset is publicly available at the 10x Genomics website (https://www.10xgenomics.com/datasets/pbmc-from-a-healthy-donor-no-cell-sorting-10-k-1-standard-1-0-0). The BMMC, MA, and Chen datasets can be downloaded from GEO under accession numbers GSE194122, GSE140203, and GSE126074, respectively. For cross-dataset generation experiments, the independent PBMC dataset can be downloaded from https://cf.10xgenomics.com/samples/cell-arc/2.0.0/pbmc_granulocyte_sorted_10k/pbmc_granulocyte_sorted_10k_web_summary.html and MA dataset can be obtained from GEO under accession number GSE140203. The BMMC-CITE dataset used for proteomics translation experiments is also available from GEO under accession number GSE194122. The human glioblastoma dataset has been deposited in the Genome Sequence Archive in National Genomics Data Center that is publicly accessible at https://ngdc.cncb.ac.cn/gsa-human (accession number HRA017237).
