# Supplementary material for: scDAU: a disentangled representation learning method for cross-modal translation in single-cell multi-omics data
Source: Bioinformatics. 2026 Aug 14;42(8):btag610. doi: 10.1093/bioinformatics/btag610 (PMC13505623; doi:10.1093/bioinformatics/btag610)
Supplement: Supplementary file 1 [file btag610_supplementary_data.pdf]

scDAU: a disentangled representation learning method for cross-modal translation in single-cell multi-omics data

Supplementary Material

Jialiang Xue[1], Xiangmei Cao[2], Junlei Zhou[1], Yaowei Cao[1], Fangyuan Shi[1], Fang Du[1], Zhenhua Yu[1,*]

[1]School of Information Engineering, Ningxia University, Yinchuan, China

[2]Basic Medical School, Ningxia Medical University, Yinchuan, China

*To whom correspondence should be addressed.

Contact: zhyu@nxu.edu.cn

Table of contents

1. Supplementary Methods 2
   1.1. Evaluation metrics 2
   1.2. Autoencoders for dimensionality reduction 2
   1.3. Implementation 3
   1.4. Single-cell sequencing of the human glioblastoma sample 3
   1.5. Cell type annotation for the human glioblastoma dataset 4
2. Supplementary Results 4
   2.1 Runtime performance analysis 4
   2.2 Feature disentanglement analysis 4
   2.3 Analysis of generated human glioblastoma data 5
   2.4 Ablation study 6
3. Supplementary Figures 8
4. Supplementary Tables 14

1. Supplementary Methods

1.1. Evaluation metrics

For scRNA-seq and scATAC-seq, we employ the Pearson Correlation Coefficient (PCC) and the area under the receiver operating characteristic curve (AUROC) to quantify the agreement between the generated data and the corresponding experimental measurements, respectively.
The PCC measures the strength and direction of a linear relationship between two continuous variables, with values ranging from −1 to 1, where 1 and −1 indicate perfect positive and negative linear correlations, respectively, and 0 denotes no linear correlation. The PCC is computed as:

$$\text{PCC} = \frac{\sum_{i=1}^{n}(x_i - \bar{x})(y_i - \bar{y})}{\sqrt{\sum_{i=1}^{n}(x_i - \bar{x})^2 \cdot \sum_{i=1}^{n}(y_i - \bar{y})^2}}$$

where *n* denotes sample size, x_i and y_i represent the values for the *i*-th sample in the generated and original data, respectively, $\bar{x}$ and $\bar{y}$ are their corresponding arithmetic means.
Single-cell RNA-seq data typically exhibit highly skewed distributions, with most genes showing low or zero expression and a small subset exhibiting high expression levels. To mitigate the effect of this skewness and better satisfy the normality assumption underlying Pearson correlation analysis, we apply a logarithmic transformation $\log(x+1)$ to the RNA expression data prior to PCC calculation. This transformation stabilizes variance and attenuates the influence of extreme outliers, thereby enhancing the accuracy and robustness of the correlation estimates. All PCC values reported in this study are computed using log-transformed expression data.
AUROC is a widely used metric for evaluating the performance of binary classifiers, reflecting a model's ability to discriminate between positive and negative instances. AUROC values range from 0 to 1, with 0.5 indicating random performance and 1.0 representing perfect discrimination. Formally, it can be computed as:

$$\text{AUROC} = \frac{1}{mn}\sum_{i=1}^{m}\sum_{j=1}^{n}\mathbf{1}(s_i^{+} > s_j^{-})$$

where *m* and *n* denote the number of positive and negative samples, s_i^{+} and s_j^{-} are the predicted scores for the *i*-th positive and *j*-th negative samples, respectively, and $\mathbf{1}(\cdot)$ is an indicator function that equals 1 if the positive score exceeds the negative score and 0 otherwise. The AUROC is then obtained by averaging over all $m \times n$ positive-negative pairs.
This metric is robust to class imbalance, which is common in genomic datasets. In single-cell multi-omics analyses, AUROC is frequently used to evaluate the fidelity of reconstructed scATAC-seq peaks, providing a quantitative measure of a model's ability to recover true biological signals.

1.2. Autoencoders for dimensionality reduction

In scDAU, two separate autoencoders were employed to perform dimensionality reduction for scRNA-seq and scATAC-seq data, respectively. The RNA encoder E_r consists of two fully connected layers: a 512-dimensional hidden layer with LeakyReLU activation, followed by a 256-dimensional latent layer. To improve model robustness and reduce overfitting, dropout was applied at two stages, with a rate of 0.5 at the input layer for data augmentation and a rate of 0.1 at the hidden layer. Batch normalization was applied after each linear transformation to stabilize training. The decoder D_r adopts a symmetric architecture, expanding the 256-

dimensional latent representation through a 512-dimensional hidden layer to reconstruct the original gene expression profile.

The ATAC autoencoder was tailored to the structural characteristics of scATAC-seq data. A key feature of this design is a per-chromosome processing mechanism that explicitly captures chromosomal organization. In the encoder E_a, the first layer applies independent linear transformations to the peak features from each chromosome, projecting each chromosome-specific input into a 32-dimensional feature space. These chromosome-level embeddings were then concatenated across all chromosomes and further compressed by a second linear layer into a 256-dimensional latent representation. The decoder D_a performs the inverse operation. It first decodes the latent vector into a fused intermediate representation, which was subsequently partitioned and projected back to reconstruct chromosome-specific peak accessibility profiles. A sigmoid activation function was applied at the output layer to model the binary nature of the scATAC-seq data. This architecture enables the model to capture both local chromosomal accessibility patterns and global cross-chromosomal regulatory information.

1.3. Implementation

All experiments were conducted on a server equipped with 128 GB RAM and four NVIDIA RTX 3090 Ti GPUs. For dimensionality reduction, the latent space dimension was set to 256. The RNA and ATAC autoencoders were trained independently with a learning rate of 0.001, a batch size of 32, and for 250 epochs. The feature decoupling module was trained with the same learning rate and batch size for 120 epochs. Finally, the cross-modal translation U-Net networks were trained with a learning rate of 0.0001 and a batch size of 32 for 20 epochs.

1.4. Single-cell sequencing of the human glioblastoma sample

Fresh human glioblastoma tissue from a patient was dispatched to Singleron Biotechnologies for simultaneous scRNA-seq and scATAC-seq. For scRNA-seq, single-cell suspensions were adjusted to 2×10^5 cells/mL in PBS (HyClone, Shanghai, China), loaded onto microfluidic devices, and were processed using the GEXSCOPE® Single-Cell RNA Library Kit and Matrix NEO® automated system (Singleron Biotechnologies). Libraries were diluted to 4 ng/μL, pooled, and sequenced on an Illumina NovaSeq 6000 platform with paired-end 150 bp reads. For scATAC-seq, libraries were constructed using the Singleron Single Cell ATAC Library Kit for SD, following standard steps including nuclear extraction, transposition, product capture, ligation, inactivation, and PCR amplification. Final scATAC-seq libraries were diluted to 5-10 nM and sequenced on an Illumina NovaSeq 6000 platform using paired-end 150 bp reads.

scRNA-seq data were processed using the CeleScope pipeline (v2.7.3; https://github.com/singleron-RD/CeleScope). Raw sequencing reads were demultiplexed and aligned to the human reference genome (Ensembl release 110). Gene-level quantification was performed using STARsolo (v2.7.11b; https://github.com/alexdobin/STAR/blob/master/docs/STARsolo.md), yielding the gene expression count matrix. scATAC-seq data were processed using the CeleScope-ATAC pipeline (v1.8.1; https://github.com/singleron-RD/CeleScope_ATAC). Reads were demultiplexed, aligned, deduplicated, and subjected to peak calling and cell calling to generate the chromatin accessibility matrix. Alignment was performed with Chromap (v0.2.6; https://github.com/haowenz/chromap/blob/master/README.md), and

peaks were identified using MACS2 (v2.2.7.1; https://github.com/macs3-project/MACS/blob/master/README.md), with the human reference genome from Ensembl release 109.

1.5. Cell type annotation for the human glioblastoma dataset

Cell clusters in the human glioblastoma scRNA-seq data were identified using Scanpy [1]. DEGs for each cluster were identified using the Wilcoxon rank-sum test, followed by preliminary immune cell screening with the CellTypist [2] automated tool to establish high-confidence cell identities. For malignant cell refinement, we manually annotated human glioblastoma subtypes based on a previous study [3].

For the human glioblastoma scATAC-seq data, cell type annotations were transferred from the scRNA-seq data using Seurat v4 [4]. Specifically, expression data of 3,000 highly variable genes were selected, and dimensionality reduction was performed by PCA; scATAC-seq peak matrices were processed using a ChromatinAssay built on the hg38 genome, followed by TF‑IDF normalization and SVD. Gene activity matrices were computed from ATAC fragment files, and canonical correlation analysis (CCA) was used to identify cross-modality anchors based on scRNA-seq variable genes. Cell type labels were then transferred using Seurat's label transfer framework.

2. Supplementary Results

2.1 Runtime performance analysis

To assess computational efficiency and scalability, we compared the runtime performance of all methods on both the PBMC and MA datasets (Tables S1–S2). Across the two datasets, scDAU demonstrated competitive training speed while maintaining relatively low RAM consumption. On the PBMC dataset, scDAU completed training in 0.82 h, outperforming scMM (1.24 h), scButterfly (1.34 h), and scDCT (7.88 h), while being only slightly slower than BABEL (0.52 h) and JAMIE (0.71 h). On the MA dataset, scDAU required 0.97 h, remaining substantially faster than scButterfly (2.40 h) and scDCT (9.45 h), and its training time is comparable to JAMIE (0.89 h) and BABEL (0.81 h). In terms of memory consumption, scDAU required 13.54 GB RAM on PBMC and 19.66 GB on MA, representing the second-lowest RAM usage on PBMC, slightly higher than JAMIE (12.81 GB), and the lowest RAM usage on MA among all compared methods. By contrast, competing approaches consumed between 26.98–81.72 GB RAM on the MA dataset. Although scDAU utilized relatively high GPU memory (12.52 GB on PBMC and 21.74 GB on MA), this increased GPU utilization enabled substantially faster training than computationally intensive methods such as scDCT. Moreover, the training time of scDAU increased only modestly from PBMC to MA (0.82 h to 0.97 h), indicating good scalability with increasing dataset size.

2.2 Feature disentanglement analysis

We performed analyses on the PBMC and MA datasets to evaluate the effectiveness of feature disentanglement. In the joint embedding space (Supplementary Fig. 2A–B), t-SNE visualization revealed a clear separation between shared and modality-specific features. The shared features (Z_{rs} and Z_{as}) exhibited substantial overlap and occupied a central region of the embedding space, indicating successful alignment of cross-modal information. In contrast, the modality-specific features (Z_{rm} and Z_{am}) formed distinct clusters that were clearly separated from both the shared features and each other, reflecting modality-specific information unique to each omics modality.

We further applied K-means clustering independently to each feature type (Supplementary Fig. 2C–D). The shared features (Z_{rs} and Z_{as}) effectively captured biologically meaningful cell-type information. In contrast, the modality-specific features (Z_{rm} and Z_{am}) exhibited highly homogeneous clustering patterns dominated by a single large cluster and showed little correspondence with cell types. Together, these results demonstrated that scDAU successfully disentangled shared biological signals from modality-specific variations, validating the effectiveness of its representation decomposition strategy.

2.3 Analysis of generated human glioblastoma data

To evaluate the biological fidelity of the human glioblastoma data generated by scDAU, we performed Gene Ontology (GO) enrichment, marker gene validation, and transcription factor motif analysis on the translated data.

GO enrichment analysis identifies biological processes that are overrepresented among cell type-specific upregulated genes. We identified cell type-specific upregulated genes using the Wilcoxon rank-sum test on log-normalized expression matrices via Scanpy [1], with an FDR < 0.05. A logFC threshold of >0.5 was applied for non-malignant subpopulations and >0.25 for malignant states, and only genes detected in ≥10 of cells within the respective subpopulation were considered. Following this, we removed genes associated with mitochondrial, ribosomal, or immunoglobulin functions, heat shock proteins, and other low-confidence genes. The remaining genes were ranked by logFC, with the top 100 (for non-malignant) and top 300 (for malignant) upregulated genes being selected for enrichment testing. Enrichment was assessed using Fisher's exact test (via GSEApy/Enrichr) [5,6] against the GO_Biological_Process_2023 database [7], with significance determined independently per subpopulation using the Benjamini-Hochberg [8] procedure.

As shown in Supplementary Fig. 4A-B, the results demonstrated that the scDAU-generated RNA data faithfully recapitulates the cell type-specific biological programs of the original scRNA-seq data at a functional level. Major cell subpopulations exhibited highly consistent patterns of enriched GO terms in both datasets, covering key processes such as tumor proliferation, regulation of neurogenesis, inflammatory response, synaptic transmission, myelination, and vascular/stromal remodeling. This indicated that the model stably preserved the core functional signatures of major cell lineages. Notably, the Malignant (Cycling) subpopulation was consistently enriched for terms related to mitotic sister chromatid segregation, mitotic spindle organization, DNA repair, and spindle assembly checkpoint signaling in both the original and translated data, demonstrating the model's effectiveness in retaining the characteristic high-proliferation program of glioma cells. Furthermore, pathways such as *maintenance of blood-brain barrier*, *vasculature development*, and *extracellular matrix organization* were clearly recovered in the generated data. This finding indicated that the model not only accurately reconstructed signals from the main tumor mass but also preserved the functional states of vascular and stromal cells within the tumor microenvironment.

We next compared marker genes derived from the generated data against established markers in the CellMark 2.0 database (Supplementary Fig. 4C-D). The comparison revealed strong concordance with established cell-type markers. For instance, elevated expression of *CKAP2L* in Cycling Malignant cells, *CD34* in Endothelial cells, and *MAL* in Oligodendrocyte cells matches known marker patterns, confirming the biological fidelity of the generated data at the level of cell-type-specific gene expression.

Finally, we leveraged the scDAU-generated ATAC data to search for significantly enriched DNA sequence motifs within differentially accessible peaks across cell types. To systematically probe chromatin accessibility features of key human glioblastoma regulators, we focused on four factors pivotal in human

glioblastomagenesis: the tumor suppressor *PTEN*, the proto-oncogene *MYC*, the signaling mediator *STAT3*, and the stemness factor *SOX2*. Sequence logos for motifs enriched for these factors were generated (Supplementary Fig. 5). This multi-faceted analysis explores chromatin accessibility patterns related to tumor suppressor inactivation, oncogene activation, aberrant signaling, and stem cell properties. The sequence logos revealed highly consistent motif patterns between the generated and original measured data, with only minor quantitative differences at a few positions. This suggested that scDAU can produce chromatin accessibility data of high biological quality from complex tumor datasets, thereby providing a reliable in silico resource for subsequent mechanistic studies.

2.4 Ablation study

To enhance the extraction of cross-modally shared semantic features during the decoupling stage, scDAU employed a shared conditional diffusion process for data reconstruction rather than a conventional shared linear decoder. To evaluate the contribution of this design choice, we conducted an ablation study in which the conditional diffusion module is replaced with a linear decoder while keeping all other components unchanged. This modification led to a consistent degradation in translation performance. As shown in Supplementary Fig. 7, on the MA dataset, the PCC for ATAC-to-RNA translation dropped from 0.823 to 0.804, and the AUROC for RNA-to-ATAC translation also showed a decrease. These results suggest that the conditional diffusion module provides a more expressive and robust mechanism for reconstructing features, thereby facilitating more accurate cross-modal translation and validating its inclusion in the scDAU architecture.

In addition to the reconstruction module, scDAU was specifically designed with a two-stage training pipeline, in which feature decoupling and modality translation were explicitly separated and optimized in distinct stages. To assess the necessity of this staged training strategy, we performed an ablation study using a joint training scheme, where feature extraction and cross-modal translation were optimized simultaneously. Under this joint training setting, translation performance deteriorated substantially across both modalities and datasets. As reported in Supplementary Fig. 7, PCC scores dropped by more than 0.3 and AUROC scores decreased by over 0.08 on both the PBMC and MA datasets. This marked performance degradation underscores the importance of separating feature decoupling and translation during training. Since feature extraction and cross-modal translation are inherently distinct tasks with different sensitivities to training dynamics, forcing joint optimization can introduce interference and suboptimal performance for both.

Reference:

[1] Wolf F A, Angerer P, Theis F J. SCANPY: large-scale single-cell gene expression data analysis[J]. Genome biology, 2018, 19(1): 15.

[2] Domínguez Conde C, Xu C, Jarvis L B, et al. Cross-tissue immune cell analysis reveals tissue-specific features in humans[J]. Science, 2022, 376(6594): eabl5197.

[3] Neftel C, Laffy J, Filbin M G, et al. An integrative model of cellular states, plasticity, and genetics for glioblastoma[J]. Cell, 2019, 178(4): 835-849. e21.

[4] Hao Y, Hao S, Andersen-Nissen E, et al. Integrated analysis of multimodal single-cell data[J]. Cell, 2021, 184(13): 3573-3587. e29.

[5] Fang Z, Liu X, Peltz G. GSEApy: a comprehensive package for performing gene set enrichment analysis in Python[J]. Bioinformatics, 2023, 39(1): btac757.

[6] Kuleshov M V, Jones M R, Rouillard A D, et al. Enrichr: a comprehensive gene set enrichment analysis web server 2016 update[J]. Nucleic acids research, 2016, 44(W1): W90-W97.

[7] Aleksander S A, Balhoff J, Carbon S, et al. The gene ontology knowledgebase in 2023[J]. Genetics, 2023, 224(1): iyad031.

[8] Benjamini Y, Hochberg Y. Controlling the false discovery rate: a practical and powerful approach to multiple testing[J]. Journal of the Royal statistical society: series B (Methodological), 1995, 57(1): 289-300.

3. Supplementary Figures

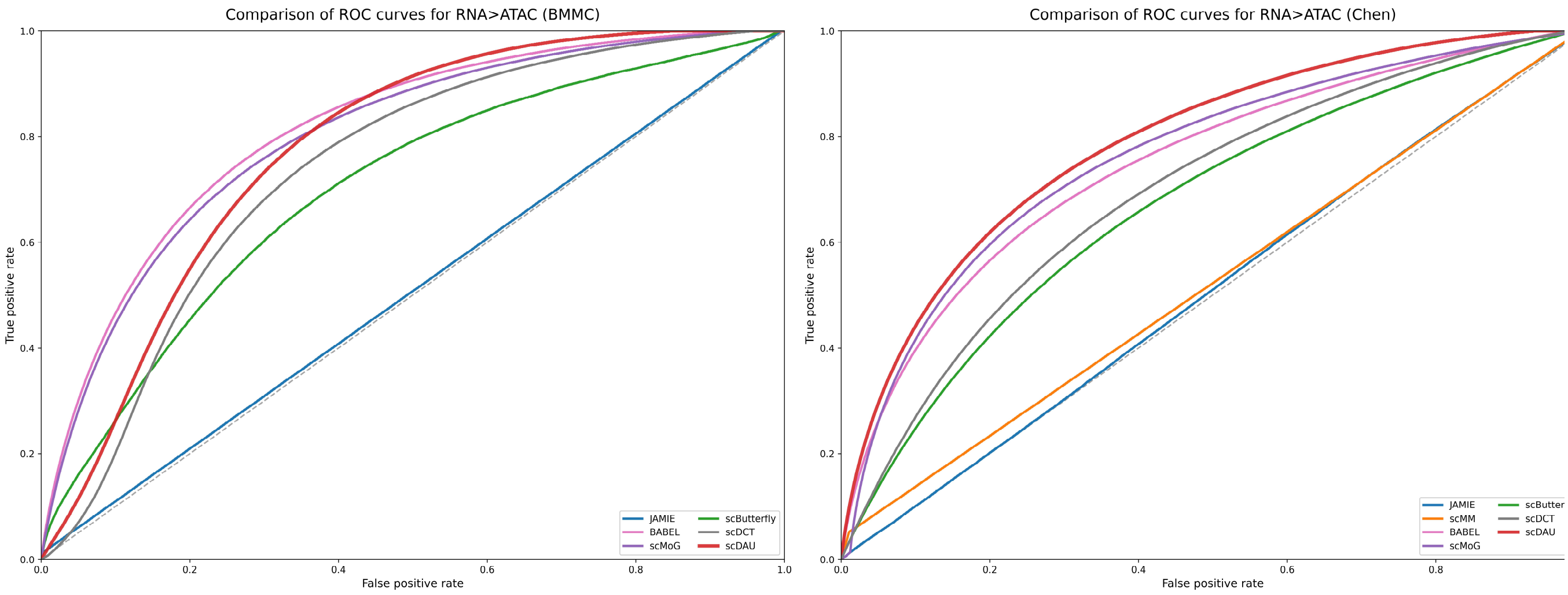

Figure S1. Visualization of RNA-to-ATAC translation performance. The area under the receiver operating characteristic curve (AUROC) is computed by comparing the generated ATAC profiles with the raw ATAC data on the BMMC and Chen datasets.

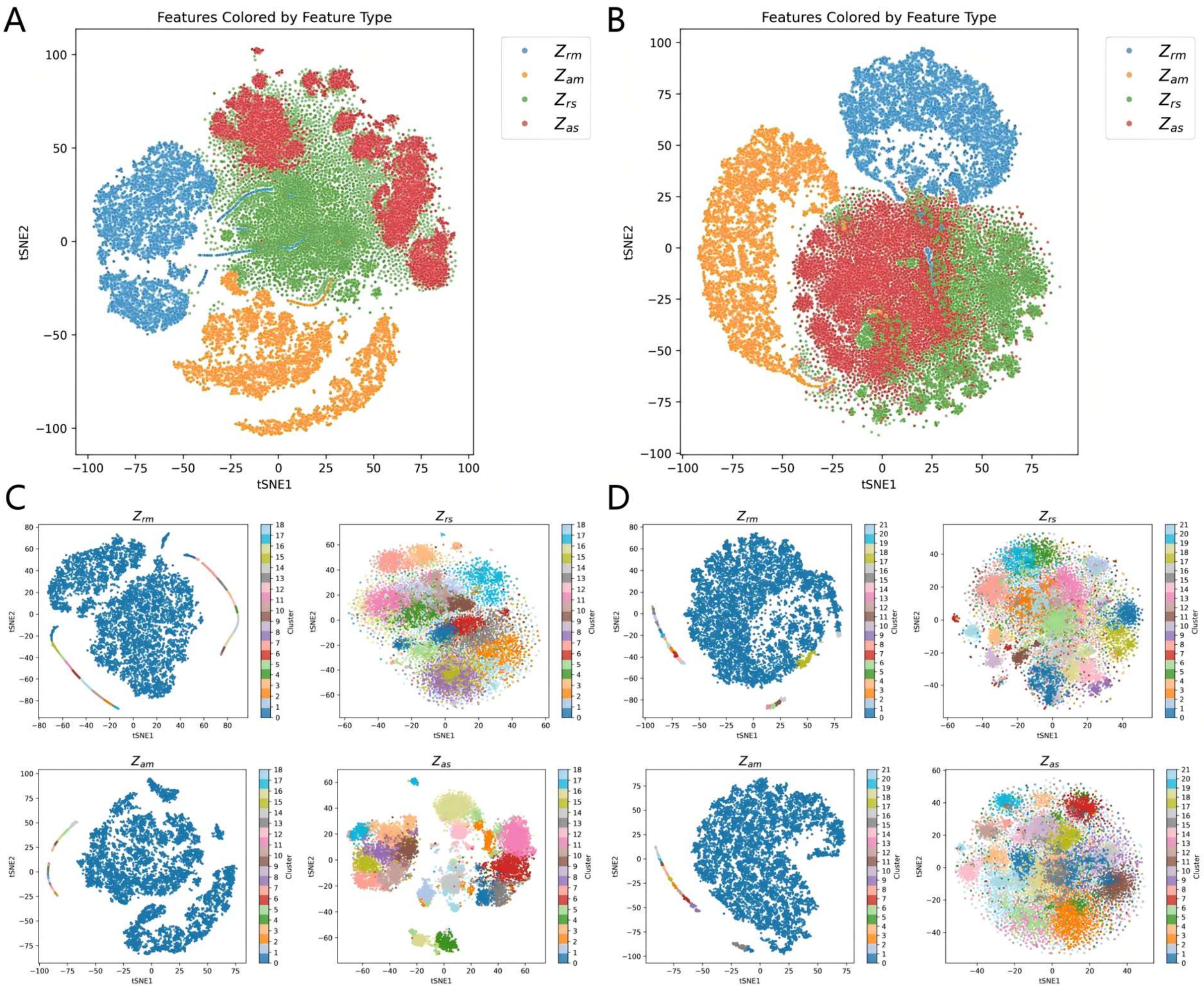

Figure S2. Visualization of disentangled latent spaces reveals distinct modality-specific and shared feature representations. (A, B) Joint t-SNE visualization of the four latent feature spaces, including RNA-specific features (Z_{rm}), ATAC-specific features (Z_{am}), and shared features (Z_{rs} and Z_{as}), for the PBMC (A) and MA (B) datasets. The results demonstrated a clear separation between modality-specific and shared representations. (C, D) K-means clustering performed independently on each latent feature space for the PBMC (C) and MA (D) datasets. Cell type-specific clustering patterns were observed predominantly in the shared feature spaces (Z_{rs} and Z_{as}), whereas modality-specific features (Z_{rm} and Z_{am}) showed limited cell type organization, indicating that biologically relevant information was primarily captured by the shared representations.

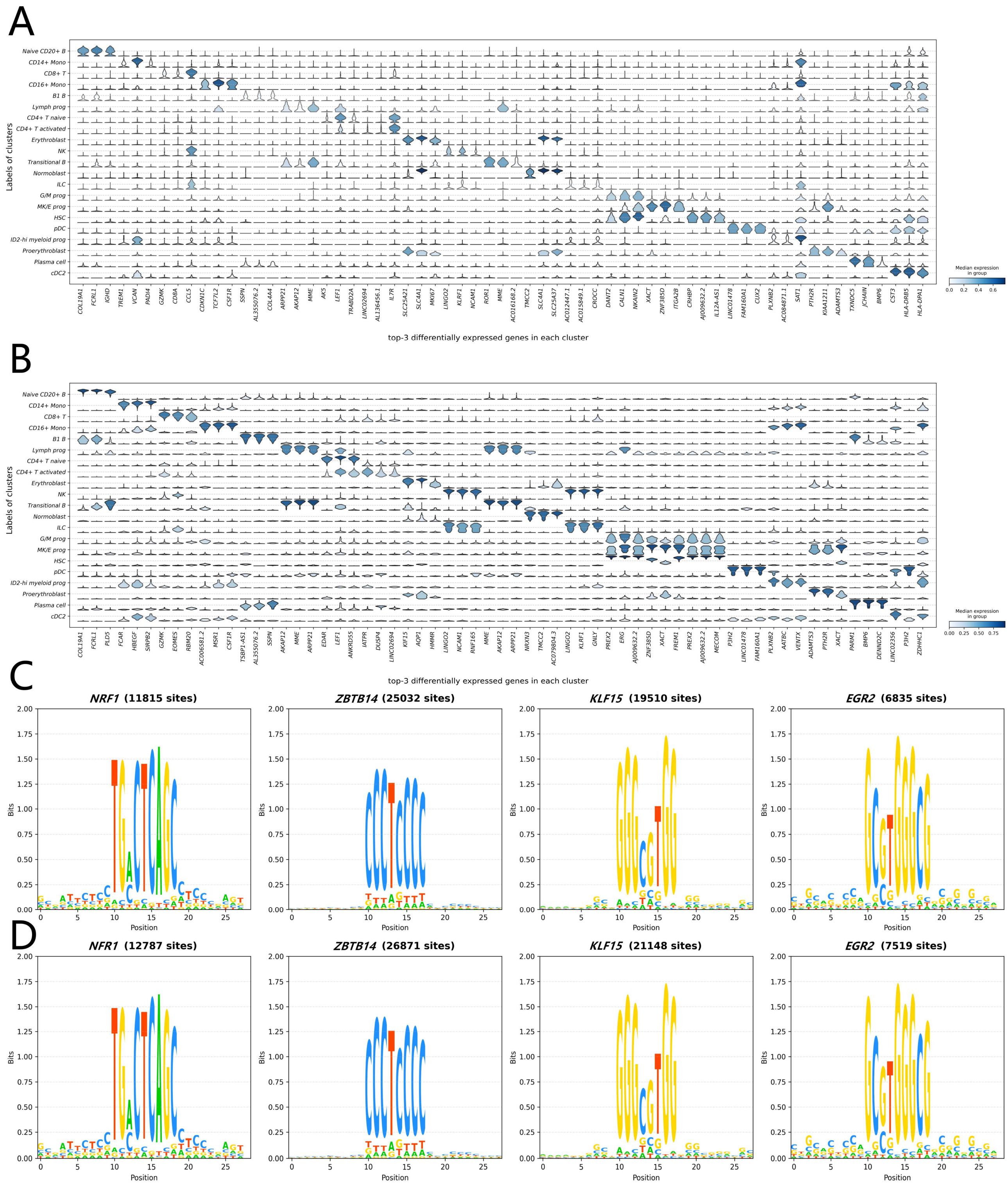

Figure S3. scDAU preserves key biological signals during cross-modal translation. (A, B) Expression patterns of differentially expressed genes (DEGs) across cell types. Stacked violin plots display the expression levels of the top three DEGs per cell type in the BMMC raw RNA dataset (A) and the scDAU-generated RNA dataset (B). (C, D) Comparison of chromatin accessibility patterns on the PBMC dataset. Representative DNA sequence motifs identified from the original ATAC-seq data (C) and the scDAU-generated ATAC-seq data (D).

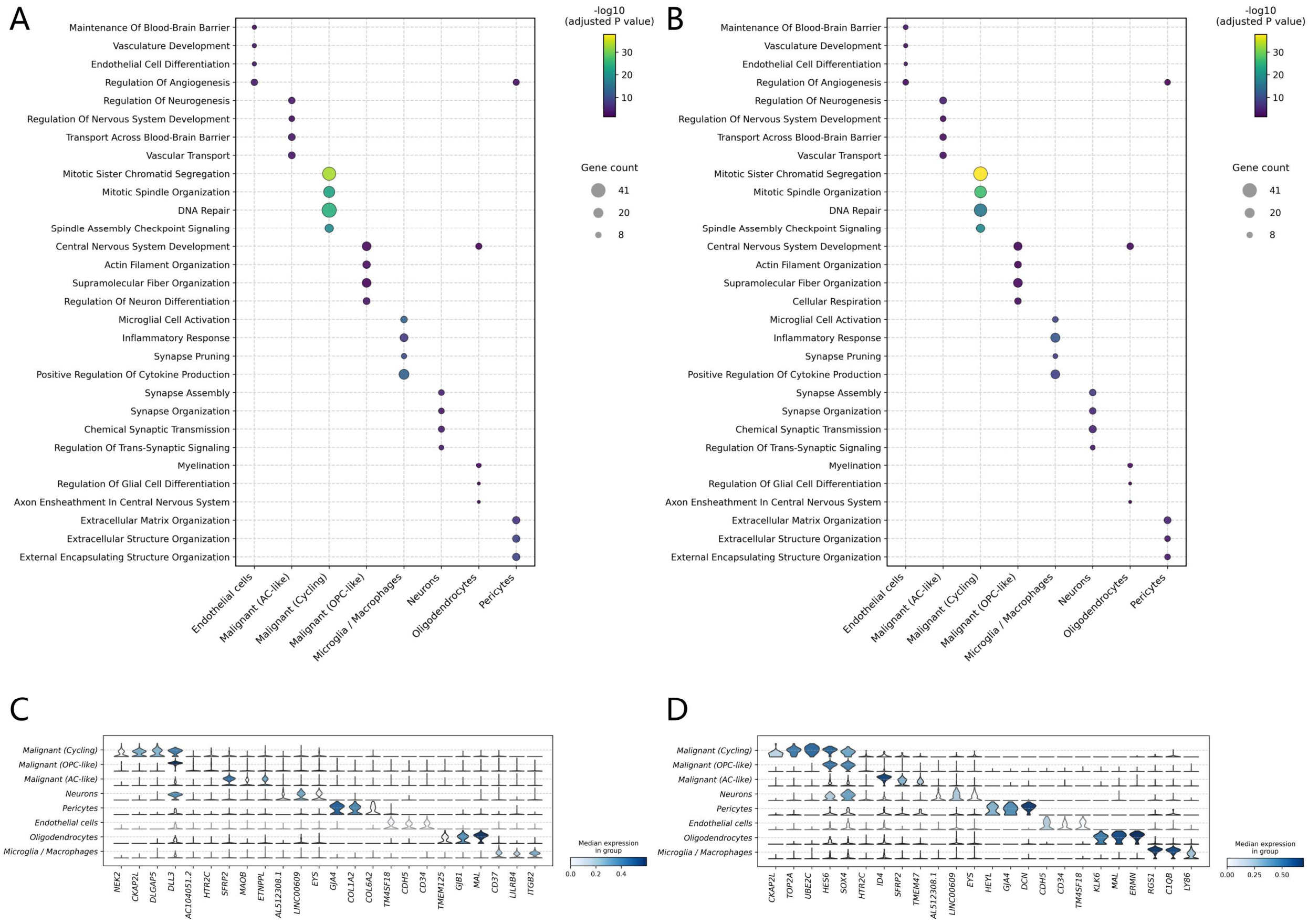

Figure S4. Go enrichment and marker gene analysis on the human glioblastoma dataset. (A) GO enrichment analysis results of raw human glioblastoma dataset. (B) GO enrichment analysis results of generated human glioblastoma dataset. (C, D) Expression patterns of differentially expressed genes (DEGs) across cell types. Stacked violin plots display the expression levels of the top three DEGs per cell type in the human glioblastoma raw RNA dataset (C) and the scDAU-generated RNA dataset (D).

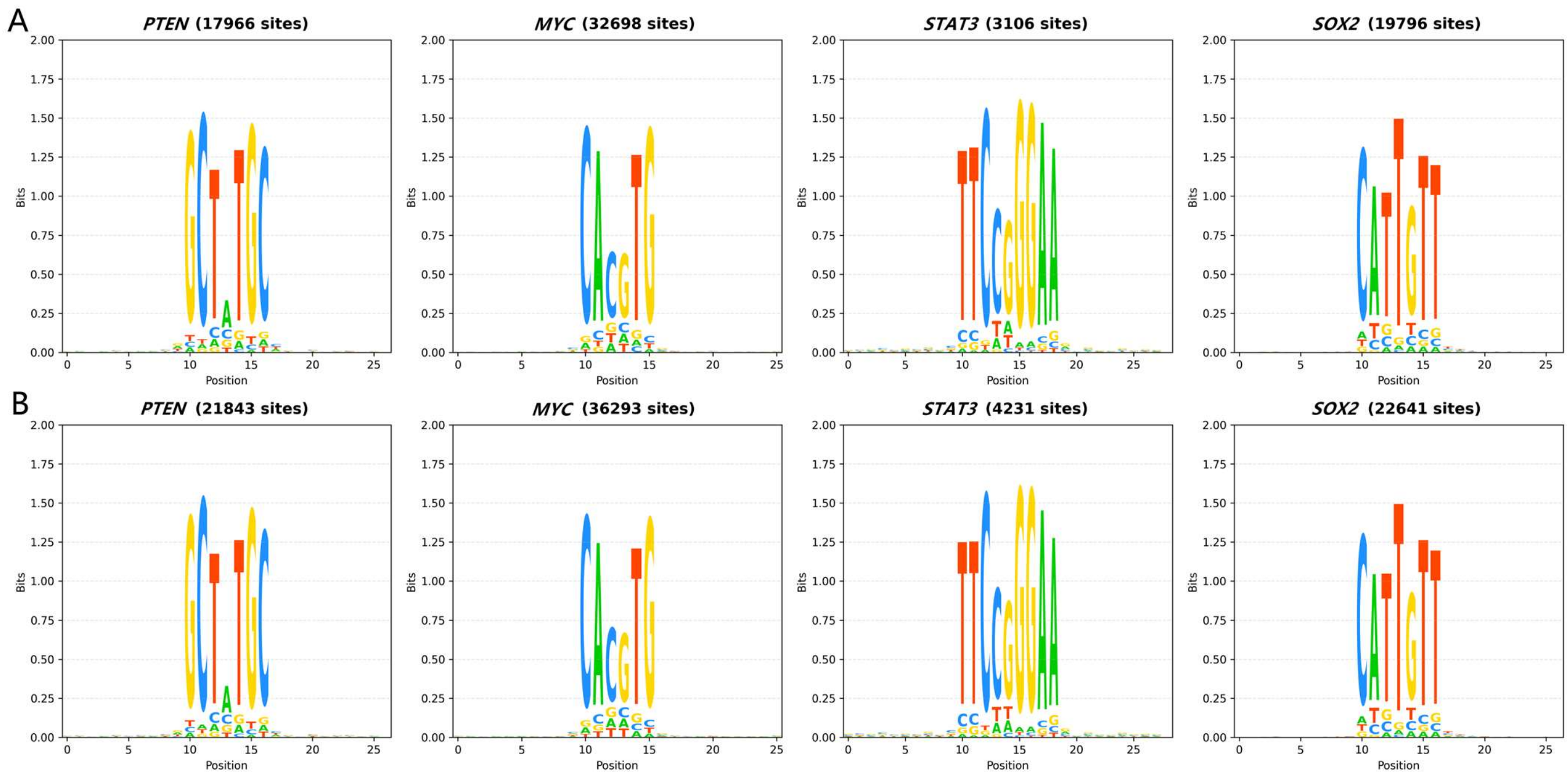

Figure S5. Comparison of chromatin accessibility patterns on the human glioblastoma dataset. Representative DNA sequence motifs identified from the original ATAC-seq data (A) and the scDAU-generated ATAC-seq data (B).

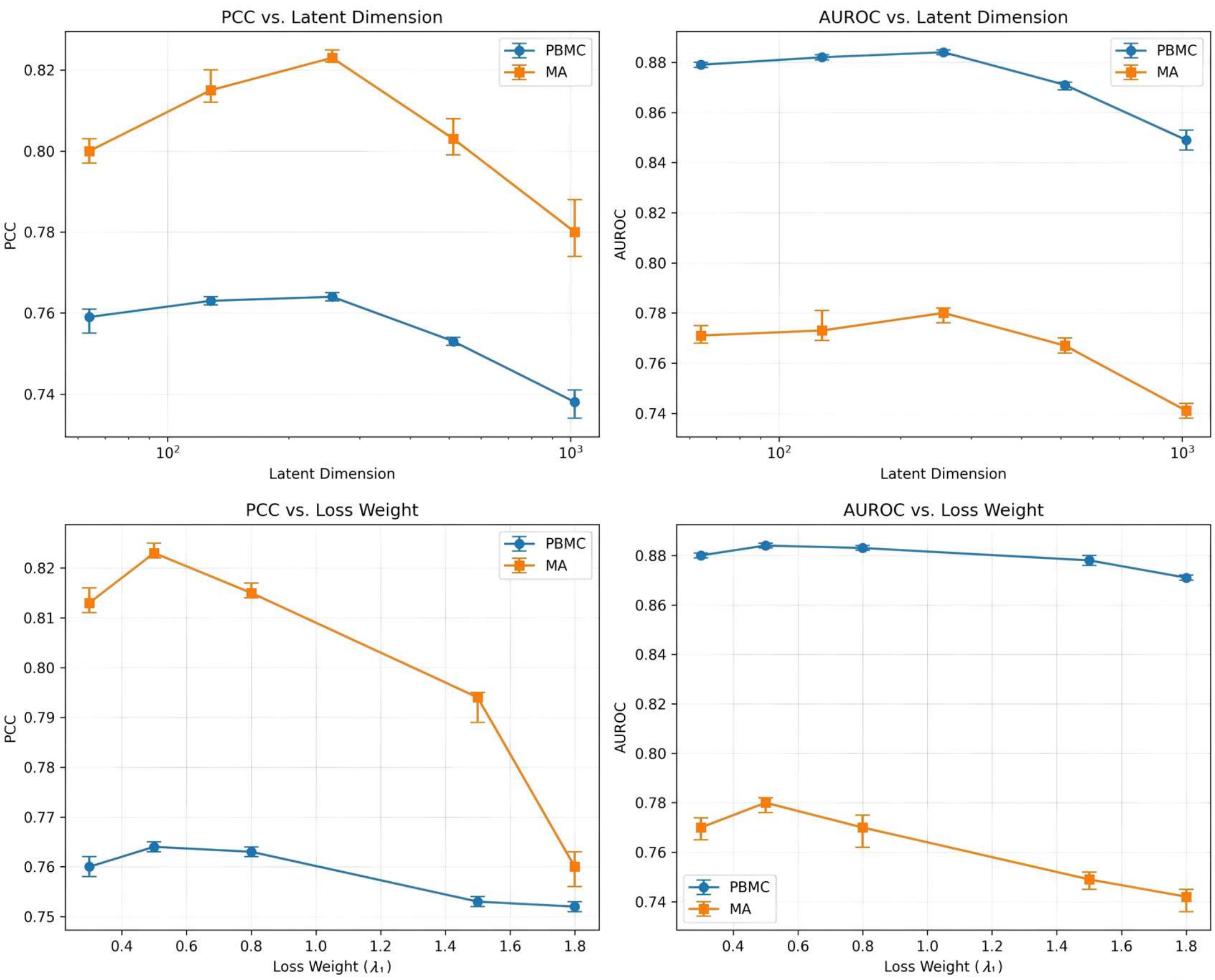

Figure S6. Hyperparameter experiments with different latent dimensions and loss weights.

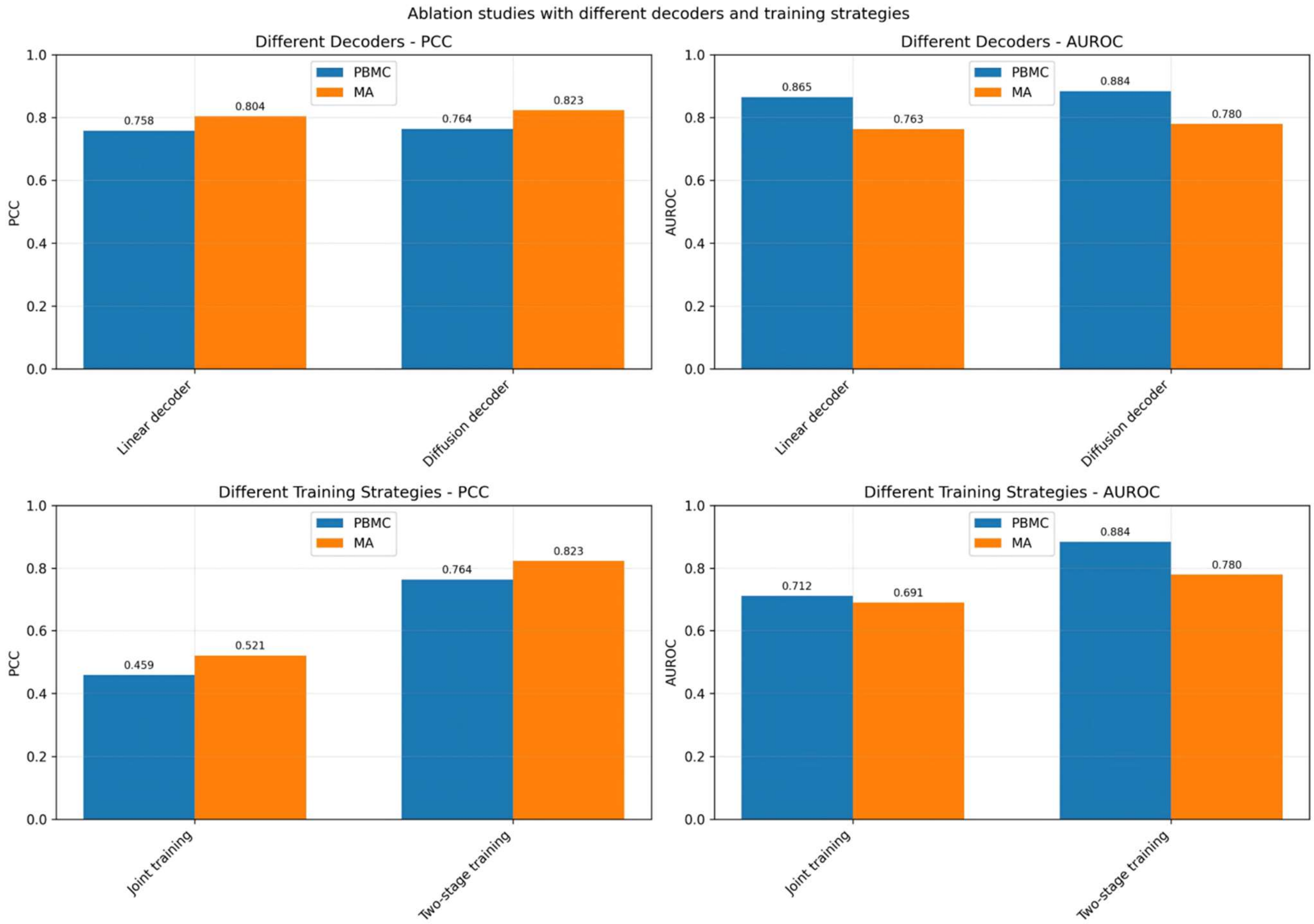

Figure S7. Ablation studies with different decoders for data reconstruction and strategies for model training.

4. Supplementary Tables

Table S1. Runtime performance analysis results on PBMC dataset.

Method*	RAM consumption (GB)	GPU memory usage (GB)	Time (hours)
JAMIE	12.81	3.66	0.71
scMM	34.94	1.87	1.24
BABEL	28.08	5.67	0.52
scMoG*	43.69	0.00	4.83
scButterfly	30.16	1.57	1.34
scDCT	16.66	11.46	7.88
scDAU	13.54	12.52	0.82

*We failed to run scMoG on GPU devices.

Table S2. Runtime performance analysis results on MA dataset.

Method*	RAM consumption (GB)	GPU memory usage (GB)	Time (hours)
JAMIE	38.02	4.45	0.89
scMM	62.21	2.50	1.71
BABEL	59.80	10.67	0.81
scMoG	81.72	0.00	10.04
scButterfly	48.45	2.09	2.40
scDCT	26.98	19.89	9.45
scDAU	19.66	21.74	0.97

*We failed to run scMoG on GPU devices.
